# Medical News and Bulletin

**Published:** 1843-04-01

**Authors:** 


### MEDICAL NEWS AND BULLETIN.

ARM-PRESENTATION IN NINE SUCCESSIVE ACCOUCHEMENTS.

This very remarkable circumstance is stated in the "Gazette des Hopitaux" to have taken place in a woman of large build, usually enjoying good health and married to a man also of a stout and vigorous frame. In her first labor the presentation was natural, and the child was born without the aid of any medical attendant. But in her nine succeeding labours, all of which took place within the next ten years, the arm almost uniformly presented; and all the children being of large size, only one was born alive. The woman died two days after her eleventh labour.—*Lond. Lancet*, Feb. 4, 1843.

EXAMINATION OF THE BODY OF MR. RICHARD CARLILE.

The well-known Mr. Richard Carlile, bookseller, late of Fleet-street, bequeathed his body for the purpose of anatomical dissection. By permission of the governors of St. Thomas's Hospital his remains were removed from his residence in Bouverie-street, Fleet-street, to that institution, and on Tuesday last there was a numerous assemblage of the friends of the deceased and members of the medical profession, in the anatomical theatre, to witness his post mortem examination. The chest and abdomen merely were opened, and the necessity that existed for the knowledge of anatomy, not only to the surgeon, but to the physician, was shown. Mr. Grainger delivered a short address on the occasion, thinking that the object of the deceased would be obtained by this simple proceeding in public, and by a statement of the motives which had actuated him in giving his remains for dissection.

The illustrious Bentham, actuated by the same benevolent feeling, had, at the close of the last century, left his body for dissection, and that at a time when the prejudice against anatomical examinations was so great that bodies were procured with the utmost difficulty. The popular feeling against dissection was, perhaps, at the present period, less than at that time, but still sufficiently strong to interfere very materially with that due supply of subjects which was so essential to the proper education of the medical student, and of such vital importance to the community at large. Mr. Carlile deserved the approbation of all friends of humanity for attempting to remove this prejudice, by leaving his remains for anatomical purposes.

Mr. Grainger dwelt on the importance of a knowledge of anatomy to every person practising medicine or surgery, and illustrated this by some well-known examples, as operations for hernia and aneurism, and of injuries to joints and fractures. He said that the present Anatomy Act was in a great degree inoperative, and that during the early part of the session many students could not obtain subjects, and idle habits were thus engendered in them, or they at least were subjected to a great loss of time. He feared that no Government would be bold enough to bring in a Bill which would remedy this defect in the present state of the public mind respecting post-mortem dissections. He remarked that he had often been placed in a most unpleasant position by being unable to obtain subjects on which country practitioners could refresh their anatomical knowledge previous to the performance of an operation. What an amount of injury to the living must result when medical practitioners were ignorant of anatomy!

He proceeded to show that the difficulties in the pursuit of anatomical knowledge were such, in this country, that no lecturer had ever yet been able to complete a course of operative surgery, properly so called, and that many students, to become proficients, were obliged to repair to France for the purpose of obtaining a necessary supply of subjects. In the case of a war this resource would be cut off.

He vindicated medical men from the charge of irreligion, and contended that medical and anatomical studies, if *properly* pursued, served to demonstrate the truth, not only of natural, but of revealed religion.—*Ibid.* Feb. 18.

THE TALK ALL ON ONE SIDE.

Desgenettes had a way peculiarly his own of conducting his inquiries respecting his patients' ailments. He would put a question, but never suffer an answer to be made. If the patient offered to speak, the professor would say, "Your interruption is neither polite nor politic; it is rude to interrupt any one who is speaking to you; and it is unwise, because while I am talking all the time I can spare elapses.—*Ibid.* Feb. 4.

FRENCH INSTITUTE.—ELECTION OF DR. RAYER.

Dr. Rayer has been elected member of the Academy of Sciences (section of agriculture) by a majority of forty-one votes in fifty-six.—*Lond. Prov. Med. Journ.* Feb. 25.

MORTALITY IN FOUNDLING HOSPITALS.

The mortality in the Foundling Hospital at Vienna was at one period 60 per cent.; in the year 1812, as high as 69 per cent. Since then, however, the mortality has gradually fallen; in 1823, only 20 per cent. died; in 1826, only 14 per cent.; in the year 1829, 23 per cent. During the last ten years the mortality never exceeded 20 per cent.—*Ibid, from Ost. Wochen.*, No. 52, 1842.

MUNIFICENT LEGACY TO ST. GEORGES HOSPITAL.

This hospital has been recently enriched by the munificent legacy of £5,000 per annum and £60,000. *Ibid*, Feb. 11, 1843.

ACADEMY OF SCIENCES, PARIS.

The candidates for the place vacant in the section of medicine by the death of M. Double, were MM. Andral, Poiseuille, Cruveilhier, Guerin, and Bourgery. On the first ballot, and of 55 voters, M. Andral obtained 42 votes; M. Guerin, 5; M. Cruveilhier, 4; M. Poiseuille, 4. M. Andral was, therefore, declared member of the Academy; and, we need not add, that the election could not have fallen on one more likely to sustain the reputation of that illustrious body.

M. Dumas was elected member in the section of physics, by a very large majority.—*Ibid.* Feb. 18.

